# Supplementary material for: Growth and Diversity of Spoiling and Foodborne Bacteria in Poultry Hamburgers in Modified Atmosphere and with Sulfites During Shelf Life
Source: Microorganisms. 2025 Mar 26;13(4):754. doi: 10.3390/microorganisms13040754 (PMC12029653; doi:10.3390/microorganisms13040754)
Supplement: Supplementary file 1 [file microorganisms-13-00754-s001.zip › microorganisms-3506975-supplementary.pdf]

Table S1. Results from the identification of 215 isolates obtained from the culture in specific conditions of the flora of poultry hamburgers from days 0 to 16 during the preservation period.

Bacterial group	Growth media	Sulphites*	Atmosphere	Sampling day	Identification
Aerobic mesophilic bacteria	TSAYE	W/O Sulphites	Unmodified	0	*Macrococcus caseolyticus*
Aerobic mesophilic bacteria	TSAYE	W/O Sulphites	Unmodified	0	*Escherichia coli*
Aerobic mesophilic bacteria	TSAYE	W/O Sulphites	Unmodified	0	*Staphylococcus lentus*
Aerobic mesophilic bacteria	TSAYE	W/O Sulphites	Unmodified	0	*Proteus mirabilis*
Aerobic mesophilic bacteria	TSAYE	W/O Sulphites	Unmodified	0	*Staphylococcus simulans*
Aerobic mesophilic bacteria	TSAYE	W/O Sulphites	Unmodified	0	*Rothia nasimurium*
Aerobic mesophilic bacteria	TSAYE	W/O Sulphites	Unmodified	0	*Corynebacterium phoceense*
Aerobic mesophilic bacteria	TSAYE	W/O Sulphites	Unmodified	0	*Staphylococcus simulans*
Aerobic mesophilic bacteria	TSAYE	W/O Sulphites	Unmodified	0	*Rothia nasimurium*
Aerobic mesophilic bacteria	TSAYE	W/O Sulphites	Unmodified	0	*Rothia nasimurium*

Aerobic mesophilic bacteria	TSAYE	W Sulphites	Unmodified	0	*Enterococcus faecalis*
Aerobic mesophilic bacteria	TSAYE	W Sulphites	Unmodified	0	*Carnobacterium maltaromaticum*
Aerobic mesophilic bacteria	TSAYE	W Sulphites	Unmodified	0	*Carnobacterium divergens*
Aerobic mesophilic bacteria	TSAYE	W Sulphites	Unmodified	0	*Carnobacterium divergens*
Aerobic mesophilic bacteria	TSAYE	W Sulphites	Unmodified	0	*Carnobacterium maltaromaticum*
Aerobic mesophilic bacteria	TSAYE	W Sulphites	Unmodified	0	*Staphylococcus saprophyticus*
Aerobic mesophilic bacteria	TSAYE	W Sulphites	Unmodified	0	*Staphylococcus cohnii*
Aerobic mesophilic bacteria	TSAYE	W Sulphites	Unmodified	0	*Carnobacterium divergens*
Aerobic mesophilic bacteria	TSAYE	W Sulphites	Unmodified	0	*Carnobacterium divergens*
Aerobic mesophilic bacteria	TSAYE	W Sulphites	Unmodified	0	*Carnobacterium divergens*
Aerobic mesophilic bacteria	TSAYE	W/O Sulphites	Modified	0	*Proteus mirabilis*
Aerobic mesophilic bacteria	TSAYE	W/O Sulphites	Modified	0	*Carnobacterium maltaromaticum*
Aerobic mesophilic bacteria	TSAYE	W/O Sulphites	Modified	0	*Proteus mirabilis*

Aerobic mesophilic bacteria	TSAYE	W/O Sulphites	Modified	0	*Proteus mirabilis*
Aerobic mesophilic bacteria	TSAYE	W/O Sulphites	Modified	0	*Proteus mirabilis*
Aerobic mesophilic bacteria	TSAYE	W/O Sulphites	Modified	0	*Proteus mirabilis*
Aerobic mesophilic bacteria	TSAYE	W/O Sulphites	Modified	0	*Rothia nasimurium*
Aerobic mesophilic bacteria	TSAYE	W/O Sulphites	Modified	0	*Microbacterium liquefaciens*
Aerobic mesophilic bacteria	TSAYE	W/O Sulphites	Modified	0	*Escherichia coli*
Aerobic mesophilic bacteria	TSAYE	W/O Sulphites	Modified	0	*Microbacterium liquefaciens*
Aerobic mesophilic bacteria	TSAYE	W/O Sulphites	Unmodified	4	*Proteus mirabilis*
Aerobic mesophilic bacteria	TSAYE	W/O Sulphites	Unmodified	4	*Proteus mirabilis*
Aerobic mesophilic bacteria	TSAYE	W/O Sulphites	Unmodified	4	*Proteus mirabilis*
Aerobic mesophilic bacteria	TSAYE	W/O Sulphites	Unmodified	4	*Proteus mirabilis*
Aerobic mesophilic bacteria	TSAYE	W/O Sulphites	Unmodified	4	*Proteus mirabilis*
Aerobic mesophilic bacteria	TSAYE	W/O Sulphites	Unmodified	4	*Escherichia coli*

Aerobic mesophilic bacteria	TSAYE	W/O Sulphites	Unmodified	4	*Proteus mirabilis*
Aerobic mesophilic bacteria	TSAYE	W/O Sulphites	Unmodified	4	*Escherichia coli*
Aerobic mesophilic bacteria	TSAYE	W/O Sulphites	Unmodified	4	*Staphylococcus simulans*
Aerobic mesophilic bacteria	TSAYE	W/O Sulphites	Unmodified	4	*Escherichia coli*
Aerobic mesophilic bacteria	TSAYE	W Sulphites	Unmodified	4	*Enterococcus faecalis*
Aerobic mesophilic bacteria	TSAYE	W Sulphites	Unmodified	4	*Pseudomonas lundensis*
Aerobic mesophilic bacteria	TSAYE	W Sulphites	Unmodified	4	*Staphylococcus simulans*
Aerobic mesophilic bacteria	TSAYE	W Sulphites	Unmodified	4	*Staphylococcus simulans*
Aerobic mesophilic bacteria	TSAYE	W Sulphites	Unmodified	4	*Carnobacterium divergens*
Aerobic mesophilic bacteria	TSAYE	W Sulphites	Unmodified	4	*Bacillus subtilis*
Aerobic mesophilic bacteria	TSAYE	W Sulphites	Unmodified	4	*Bacillus subtilis*
Aerobic mesophilic bacteria	TSAYE	W Sulphites	Unmodified	4	*Bacillus subtilis*
Aerobic mesophilic bacteria	TSAYE	W Sulphites	Unmodified	4	*Bacillus subtilis*

Aerobic mesophilic bacteria	TSAYE	W Sulphites	Unmodified	4	*Bacillus subtilis*
Aerobic mesophilic bacteria	TSAYE	W/O Sulphites	Modified	4	*Staphylococcus cohnii*
Aerobic mesophilic bacteria	TSAYE	W/O Sulphites	Modified	4	*Staphylococcus simulans*
Aerobic mesophilic bacteria	TSAYE	W/O Sulphites	Modified	4	*Staphylococcus simulans*
Aerobic mesophilic bacteria	TSAYE	W/O Sulphites	Modified	4	*Rothia nasimurium*
Aerobic mesophilic bacteria	TSAYE	W/O Sulphites	Modified	4	*Rothia nasimurium*
Aerobic mesophilic bacteria	TSAYE	W/O Sulphites	Modified	4	*Escherichia coli*
Aerobic mesophilic bacteria	TSAYE	W/O Sulphites	Modified	4	*Staphylococcus arlettae*
Aerobic mesophilic bacteria	TSAYE	W/O Sulphites	Modified	4	*Escherichia coli*
Aerobic mesophilic bacteria	TSAYE	W/O Sulphites	Modified	4	*Bacillus pumilus*
Aerobic mesophilic bacteria	TSAYE	W/O Sulphites	Modified	4	*Bacillus subtilis*
Aerobic mesophilic bacteria	TSAYE	W/O Sulphites	Unmodified	8	*Carnobacterium divergens*
Aerobic mesophilic bacteria	TSAYE	W/O Sulphites	Unmodified	8	*Carnobacterium divergens*

Aerobic mesophilic bacteria	TSAYE	W/O Sulphites	Unmodified	8	*Carnobacterium divergens*
Aerobic mesophilic bacteria	TSAYE	W/O Sulphites	Unmodified	8	*Carnobacterium maltaromaticum*
Aerobic mesophilic bacteria	TSAYE	W/O Sulphites	Unmodified	8	*Carnobacterium divergens*
Aerobic mesophilic bacteria	TSAYE	W/O Sulphites	Unmodified	8	*Bacillus subtilis*
Aerobic mesophilic bacteria	TSAYE	W/O Sulphites	Unmodified	8	*Bacillus subtilis*
Aerobic mesophilic bacteria	TSAYE	W/O Sulphites	Unmodified	8	*Bacillus subtilis*
Aerobic mesophilic bacteria	TSAYE	W/O Sulphites	Unmodified	8	*Bacillus subtilis*
Aerobic mesophilic bacteria	TSAYE	W/O Sulphites	Unmodified	8	*Bacillus subtilis*
Aerobic mesophilic bacteria	TSAYE	W Sulphites	Unmodified	8	*Carnobacterium divergens*
Aerobic mesophilic bacteria	TSAYE	W Sulphites	Unmodified	8	*Carnobacterium divergens*
Aerobic mesophilic bacteria	TSAYE	W Sulphites	Unmodified	8	*Carnobacterium divergens*
Aerobic mesophilic bacteria	TSAYE	W Sulphites	Unmodified	8	*Carnobacterium divergens*
Aerobic mesophilic bacteria	TSAYE	W Sulphites	Unmodified	8	*Carnobacterium divergens*

Aerobic mesophilic bacteria	TSAYE	W Sulphites	Unmodified	8	*Proteus mirabilis*
Aerobic mesophilic bacteria	TSAYE	W Sulphites	Unmodified	8	*Carnobacterium divergens*
Aerobic mesophilic bacteria	TSAYE	W Sulphites	Unmodified	8	*Carnobacterium divergens*
Aerobic mesophilic bacteria	TSAYE	W Sulphites	Unmodified	8	*Carnobacterium divergens*
Aerobic mesophilic bacteria	TSAYE	W Sulphites	Unmodified	8	*Carnobacterium divergens*
Aerobic mesophilic bacteria	TSAYE	W/O Sulphites	Modified	8	*Bacillus atrophaeus*
Aerobic mesophilic bacteria	TSAYE	W/O Sulphites	Modified	8	*Carnobacterium divergens*
Aerobic mesophilic bacteria	TSAYE	W/O Sulphites	Modified	8	*Escherichia coli*
Aerobic mesophilic bacteria	TSAYE	W/O Sulphites	Modified	8	*Carnobacterium divergens*
Aerobic mesophilic bacteria	TSAYE	W/O Sulphites	Modified	8	*Bacillus amyloliquefaciens*
Aerobic mesophilic bacteria	TSAYE	W/O Sulphites	Modified	8	*Staphylococcus epidermidis*
Aerobic mesophilic bacteria	TSAYE	W/O Sulphites	Modified	8	*Staphylococcus epidermidis*
Aerobic mesophilic bacteria	TSAYE	W/O Sulphites	Modified	8	*Carnobacterium maltaromaticum*

Aerobic mesophilic bacteria	TSAYE	W/O Sulphites	Modified	8	*Carnobacterium divergens*
Aerobic mesophilic bacteria	TSAYE	W/O Sulphites	Modified	8	*Carnobacterium divergens*
Aerobic mesophilic bacteria	TSAYE	W/O Sulphites	Unmodified	16	*Carnobacterium divergens*
Aerobic mesophilic bacteria	TSAYE	W/O Sulphites	Unmodified	16	*Carnobacterium divergens*
Aerobic mesophilic bacteria	TSAYE	W/O Sulphites	Unmodified	16	*Carnobacterium divergens*
Aerobic mesophilic bacteria	TSAYE	W/O Sulphites	Unmodified	16	*Carnobacterium maltaromaticum*
Aerobic mesophilic bacteria	TSAYE	W/O Sulphites	Unmodified	16	*Carnobacterium divergens*
Aerobic mesophilic bacteria	TSAYE	W/O Sulphites	Unmodified	16	*Carnobacterium maltaromaticum*
Aerobic mesophilic bacteria	TSAYE	W/O Sulphites	Unmodified	16	*Carnobacterium maltaromaticum*
Aerobic mesophilic bacteria	TSAYE	W/O Sulphites	Unmodified	16	*Carnobacterium divergens*
Aerobic mesophilic bacteria	TSAYE	W/O Sulphites	Unmodified	16	*Leuconostoc mesenteroides*
Aerobic mesophilic bacteria	TSAYE	W/O Sulphites	Unmodified	16	*Kurthia zopfii*
Aerobic mesophilic bacteria	TSAYE	W Sulphites	Unmodified	16	*Carnobacterium maltaromaticum*

Aerobic mesophilic bacteria	TSAYE	W Sulphites	Unmodified	16	*Carnobacterium divergens*
Aerobic mesophilic bacteria	TSAYE	W Sulphites	Unmodified	16	*Carnobacterium divergens*
Aerobic mesophilic bacteria	TSAYE	W Sulphites	Unmodified	16	*Carnobacterium divergens*
Aerobic mesophilic bacteria	TSAYE	W Sulphites	Unmodified	16	*Carnobacterium divergens*
Aerobic mesophilic bacteria	TSAYE	W Sulphites	Unmodified	16	*Carnobacterium divergens*
Aerobic mesophilic bacteria	TSAYE	W Sulphites	Unmodified	16	*Carnobacterium divergens*
Aerobic mesophilic bacteria	TSAYE	W Sulphites	Unmodified	16	*Carnobacterium divergens*
Aerobic mesophilic bacteria	TSAYE	W Sulphites	Unmodified	16	*Carnobacterium divergens*
Aerobic mesophilic bacteria	TSAYE	W Sulphites	Unmodified	16	*Carnobacterium divergens*
Aerobic mesophilic bacteria	TSAYE	W/O Sulphites	Modified	16	*Carnobacterium divergens*
Aerobic mesophilic bacteria	TSAYE	W/O Sulphites	Modified	16	*Carnobacterium divergens*
Aerobic mesophilic bacteria	TSAYE	W/O Sulphites	Modified	16	*Enterobacter cloacae*
Aerobic mesophilic bacteria	TSAYE	W/O Sulphites	Modified	16	*Enterobacter cloacae*

Aerobic mesophilic bacteria	TSAYE	W/O Sulphites	Modified	16	*Carnobacterium divergens*
Aerobic mesophilic bacteria	TSAYE	W/O Sulphites	Modified	16	*Leuconostoc mesenteroides*
Aerobic mesophilic bacteria	TSAYE	W/O Sulphites	Modified	16	*Carnobacterium divergens*
Aerobic mesophilic bacteria	TSAYE	W/O Sulphites	Modified	16	*Enterobacter kobei*
Aerobic mesophilic bacteria	TSAYE	W/O Sulphites	Modified	16	*Carnobacterium divergens*
Aerobic mesophilic bacteria	TSAYE	W/O Sulphites	Modified	16	*Carnobacterium divergens*
Enterobacteriaceae	VRBG	W Sulphites	Unmodified	0	*Pseudomonas lundensis*
Enterobacteriaceae	VRBG	W Sulphites	Unmodified	0	*Pseudomonas lundensis*
Enterobacteriaceae	VRBG	W Sulphites	Unmodified	0	*Staphylococcus epidermidis*
Enterobacteriaceae	VRBG	W Sulphites	Unmodified	0	*Pseudomonas lundensis*
Enterobacteriaceae	VRBG	W Sulphites	Unmodified	4	*Serratia liquefaciens*
Enterobacteriaceae	VRBG	W Sulphites	Unmodified	4	*Pseudomonas lundensis*
Enterobacteriaceae	VRBG	W Sulphites	Unmodified	4	*Citrobacter freundii*
Enterobacteriaceae	VRBG	W Sulphites	Unmodified	4	*Serratia liquefaciens*
Enterobacteriaceae	VRBG	W Sulphites	Unmodified	4	*Escherichia coli*
Enterobacteriaceae	VRBG	W/O Sulphites	Modified	4	*Escherichia coli*

Enterobacteriaceae	VRBG	W/O Sulphites	Modified	4	*Escherichia coli*
Enterobacteriaceae	VRBG	W/O Sulphites	Modified	4	*Escherichia coli*
Enterobacteriaceae	VRBG	W/O Sulphites	Modified	4	*Escherichia coli*
Enterobacteriaceae	VRBG	W/O Sulphites	Modified	4	*Hafnia alvei*
Enterobacteriaceae	VRBG	W/O Sulphites	Unmodified	16	*Enterobacter cloacae*
Enterobacteriaceae	VRBG	W/O Sulphites	Unmodified	16	*Enterobacter cloacae*
Enterobacteriaceae	VRBG	W/O Sulphites	Unmodified	16	*Enterobacter asburiae*
Enterobacteriaceae	VRBG	W/O Sulphites	Unmodified	16	*Enterobacter kobei*
Enterobacteriaceae	VRBG	W/O Sulphites	Unmodified	16	*Enterobacter kobei*
Enterobacteriaceae	VRBG	W/O Sulphites	Unmodified	16	*Enterobacter kobei*
Enterobacteriaceae	VRBG	W/O Sulphites	Unmodified	16	*Enterobacter kobei*
Enterobacteriaceae	VRBG	W/O Sulphites	Unmodified	16	*Enterobacter kobei*
Enterobacteriaceae	VRBG	W/O Sulphites	Unmodified	16	*Enterobacter kobei*
Enterobacteriaceae	VRBG	W/O Sulphites	Unmodified	16	*Enterobacter kobei*
Enterobacteriaceae	VRBG	W Sulphites	Unmodified	16	*Enterobacter kobei*
Enterobacteriaceae	VRBG	W Sulphites	Unmodified	16	*Hafnia alvei*
Enterobacteriaceae	VRBG	W Sulphites	Unmodified	16	*Hafnia alvei*
Enterobacteriaceae	VRBG	W Sulphites	Unmodified	16	*Hafnia alvei*

Enterobacteriaceae	VRBG	W Sulphites	Unmodified	16	*Hafnia alvei*
Enterobacteriaceae	VRBG	W Sulphites	Unmodified	16	*Enterobacter kobei*
Enterobacteriaceae	VRBG	W Sulphites	Unmodified	16	*Enterobacter cloacae*
Enterobacteriaceae	VRBG	W Sulphites	Unmodified	16	*Enterobacter kobei*
Enterobacteriaceae	VRBG	W Sulphites	Unmodified	16	*Enterobacter bugandensis*
Enterobacteriaceae	VRBG	W Sulphites	Unmodified	16	*Enterobacter cloacae*
Enterobacteriaceae	VRBG	W/O Sulphites	Modified	16	*Enterobacter kobei*
Enterobacteriaceae	VRBG	W/O Sulphites	Modified	16	*Enterobacter kobei*
Enterobacteriaceae	VRBG	W/O Sulphites	Modified	16	*Enterobacter kobei*
Enterobacteriaceae	VRBG	W/O Sulphites	Modified	16	*Enterobacter cloacae*
Enterobacteriaceae	VRBG	W/O Sulphites	Modified	16	*Enterobacter cloacae*
Enterobacteriaceae	VRBG	W/O Sulphites	Modified	16	*Enterobacter kobei*
Enterobacteriaceae	VRBG	W/O Sulphites	Modified	16	*Enterobacter kobei*
Enterobacteriaceae	VRBG	W/O Sulphites	Modified	16	*Enterobacter kobei*
Enterobacteriaceae	VRBG	W/O Sulphites	Modified	16	*Enterobacter kobei*
Pseudomonas spp.	CFC	W/O Sulphites	Modified	0	*Pseudomonas aeruginosa*
Pseudomonas spp.	CFC	W/O Sulphites	Modified	0	*Pseudomonas aeruginosa*
Pseudomonas spp.	CFC	W/O Sulphites	Modified	4	*Pseudomonas aeruginosa*

Pseudomonas spp.	CFC	W Sulphites	Unmodified	0	*Pseudomonas aeruginosa*
Pseudomonas spp.	CFC	W Sulphites	Unmodified	0	*Pseudomonas putida*
Pseudomonas spp.	CFC	W Sulphites	Unmodified	0	*Citrobacter freundii*
Pseudomonas spp.	CFC	W Sulphites	Unmodified	0	*Citrobacter freundii*
Pseudomonas spp.	CFC	W Sulphites	Unmodified	0	*Pseudomonas putida*
Pseudomonas spp.	CFC	W/O Sulphites	Unmodified	0	*Citrobacter freundii*
Pseudomonas spp.	CFC	W/O Sulphites	Unmodified	0	*Citrobacter freundii*
Pseudomonas spp.	CFC	W/O Sulphites	Unmodified	0	*Pseudomonas putida*
Pseudomonas spp.	CFC	W/O Sulphites	Unmodified	0	*Citrobacter freundii*
Pseudomonas spp.	CFC	W/O Sulphites	Unmodified	0	*Citrobacter freundii*
Pseudomonas spp.	CFC	W/O Sulphites	Modified	0	*Citrobacter freundii*
Pseudomonas spp.	CFC	W/O Sulphites	Modified	0	*Proteus mirabilis*
Pseudomonas spp.	CFC	W/O Sulphites	Modified	0	*Pseudomonas lundensis*
Pseudomonas spp.	CFC	W/O Sulphites	Modified	0	*Pseudomonas putida*
Pseudomonas spp.	CFC	W/O Sulphites	Modified	0	*Citrobacter freundii*
Listeria spp.	OCLA	W/O Sulphites	Modified	0	*Listeria monocytogenes*
Listeria spp.	OCLA	W/O Sulphites	Modified	0	*Listeria monocytogenes*
Listeria spp.	OCLA	W/O Sulphites	Modified	0	*Pseudomonas lundensis*

Listeria spp.	OCLA	W/O Sulphites	Modified	0	*Rothia nasimurium*
Listeria spp.	OCLA	W/O Sulphites	Unmodified	8	*Rothia nasimurium*
Listeria spp.	OCLA	W/O Sulphites	Unmodified	8	*Rothia nasimurium*
Salmonella spp.	XLD	W Sulphites	Unmodified	0	*Staphylococcus cohnii*
Salmonella spp.	XLD	W Sulphites	Unmodified	0	*Pseudomonas putida*
Salmonella spp.	XLD	W Sulphites	Unmodified	0	*Staphylococcus cohnii*
Salmonella spp.	XLD	W Sulphites	Unmodified	0	*Kocuria rhizophila*
Salmonella spp.	XLD	W Sulphites	Unmodified	0	*Escherichia coli*
Salmonella spp.	XLD	W/O Sulphites	Modified	8	*Rothia nasimurium*
Salmonella spp.	XLD	W/O Sulphites	Modified	8	*Escherichia coli*
Salmonella spp.	XLD	W/O Sulphites	Modified	8	*Enterococcus casseliflavus*
Salmonella spp.	XLD	W/O Sulphites	Modified	8	*Micrococcus sp*
Salmonella spp.	XLD	W/O Sulphites	Modified	8	*Enterococcus casseliflavus*
Salmonella spp.	XLD	W/O Sulphites	Unmodified	16	*Staphylococcus vitulinus*
Salmonella spp.	XLD	W/O Sulphites	Unmodified	16	*Escherichia coli*
Salmonella spp.	XLD	W/O Sulphites	Unmodified	16	*Staphylococcus vitulinus*
Salmonella spp.	XLD	W/O Sulphites	Unmodified	16	*Staphylococcus vitulinus*
Salmonella spp.	XLD	W/O Sulphites	Unmodified	16	*Bacillus subtilis*

Salmonella spp.	XLD	W Sulphites	Unmodified	16	*Hafnia alvei*
Salmonella spp.	XLD	W Sulphites	Unmodified	16	*Hafnia alvei*
Salmonella spp.	XLD	W Sulphites	Unmodified	16	*Hafnia alvei*
Salmonella spp.	XLD	W Sulphites	Unmodified	16	*Hafnia alvei*
Salmonella spp.	XLD	W Sulphites	Unmodified	16	*Serratia grimesii*
Salmonella spp.	XLD	W Sulphites	Unmodified	16	*Escherichia coli*
Salmonella spp.	XLD	W/O Sulphites	Modified	16	*Leuconostoc mesenteroides*
Salmonella spp.	XLD	W/O Sulphites	Modified	16	*Enterobacter kobei*
Salmonella spp.	XLD	W/O Sulphites	Modified	16	*Enterobacter kobei*
Salmonella spp.	XLD	W/O Sulphites	Modified	16	*Carnobacterium divergens*
Salmonella spp.	XLD	W/O Sulphites	Modified	16	*Leuconostoc citreum*
Salmonella spp.	XLD	W/O Sulphites	Modified	16	*Carnobacterium divergens*

*W/O (Without): Hamburgers without sulphites. W (With): Hamburgers with 5 mg/kg of sulphites.
